# Abiotic environmental factors drive the diversity, compositional dynamics and habitat preference of ectomycorrhizal fungi in Pannonian forest types

**DOI:** 10.3389/fmicb.2022.1007935

**Published:** 2022-10-12

**Authors:** József Geml, Carla Mota Leal, Richárd Nagy, József Sulyok

**Affiliations:** ^1^ELKH-EKKE Lendület Environmental Microbiome Research Group, Eszterházy Károly Catholic University, Eger, Hungary; ^2^Research and Development Centre, Eszterházy Károly Catholic University, Eger, Hungary; ^3^Biodiversity Protection Group, Bükk National Park Directorate, Eger, Hungary

**Keywords:** Basidiomycota, community assembly, forest ecology, habitat partitioning, ITS rDNA

## Abstract

Ectomycorrhizal (ECM) fungi are among the most diverse and dominant fungal groups in temperate forests and are crucial for ecosystem functioning of forests and their resilience toward disturbance. We carried out DNA metabarcoding of ECM fungi from soil samples taken at 62 sites in the Bükk Mountains in northern Hungary. The selected sampling sites represent the characteristic Pannonian forest types distributed along elevation (i.e., temperature), pH and slope aspect gradients. We compared richness and community composition of ECM fungi among forest types and explored relationships among environmental variables and ECM fungal alpha and beta diversity. The DNA sequence data generated in this study indicated strong correlations between fungal community composition and environmental variables, particularly with pH and soil moisture, with many ECM fungi showing preference for specific zonal, topographic or edaphic forest types. Several ECM fungal genera showed significant differences in richness among forest types and exhibited strong compositional differences mostly driven by differences in environmental factors. Despite the relatively high proportions of compositional variance explained by the tested environmental variables, a large proportion of the compositional variance remained unexplained, indicating that both niche (environmental filtering) and neutral (stochastic) processes shape ECM fungal community composition at landscape level. Our work provides unprecedented insights into the diversity, landscape-level distribution, and habitat preferences of ECM fungi in the Pannonian forests of Northern Hungary.

## Introduction

Topography and the physico-chemical properties of soil are among the most influential landscape-level drivers of biological communities in terrestrial ecosystems. With respect to topography, elevation and slope aspect are of particular importance, because they directly influence mesoclimatic conditions that, together with the geological history of the given site, drive several soil chemical processes, and can limit the primary productivity and the establishment of species depending on their ecological niches (Rosenberg et al., 1983; Rorison et al., 1986; McCune and Keon, 2002; Fekedulegn et al., 2003; Geml et al., 2014a,b, 2022; Gilliam et al., 2014). Despite differences in latitudinal trends in diversity, plants and fungi generally show similar levels of community structuring among biomes, biogeographic regions and landscape-level habitat types (Geml et al., 2012, 2017; Tedersoo et al., 2014; Větrovský et al., 2019; Adamo et al., 2021; Boekhout et al., 2022). According to macroecological studies of Tedersoo et al. (2014) and Větrovský et al. (2019), fungal diversity and distribution at global scales primarily are driven by climatic factors, e.g., mean annual temperature and precipitation, edaphic factors, particularly pH, as well as by dispersal limitation. There is less information on the landscape-level compositional dynamics of soil fungi, but the emerging trend from the handful of published studies is that fungal community composition at landscape scale are driven mostly by the same environmental factors as at global scales, namely, soil pH, temperature, available moisture, with the influence of individual nutrients often being dependent on habitat type (Grau et al., 2017; Geml, 2019; Geml et al., 2021).

Our study is focused on the Pannonian biogeographic region, which is unique in Europe, partly because it is a meeting point for species characteristic of distinct biogeographic regions, such as sub-Mediterranean, Pontic, Balkanian, continental, Atlantic, and Carpathian floristic and faunistic elements, and partly because of Pannonian endemics (Suba, 1983; Vojtkó, 2002; Sundseth, 2009; Vojtkó et al., 2010; Fekete et al., 2016). The region of study is located in the western half of the Bükk Mountains, a section of the mountain chain of the Északi-középhegység (North Hungarian Mountains). The geology of the region is complex, with numerous types of Paleozoic, Mesozoic, and Cenozoic calcareous, volcanic and igneous rocks appearing near the surface as a mosaic (Pelikán, 2010). Due to this geological and topographic complexity that creates a broad spectrum of edaphic and mesoclimatic conditions, the region is characterized by high habitat diversity. This is particularly true for the Bükk Mountains, as indicated by the high number of coenological vegetation types, including numerous forest and grassland communities distributed along temperature, moisture, and pH gradients (Suba, 1983; Vojtkó, 2002; Vojtkó et al., 2010).

Ectomycorrhizal (ECM) fungi are among the most prominent fungal groups in temperate forests and are key symbiotic partners of most forest trees native to the Pannonian region, particularly the ones that dominate the landscape in the study region, e.g., various species of oaks (*Quercus* spp.), beech (*Fagus sylvatica*), hornbeam (*Carpinus betulus*), birch (*Betula pendula*), as well as several species of linden (*Tilia* spp.) and whitebeam (*Sorbus* spp.). With respect to basidiomycetes, to which the majority of ECM fungi belong, the current knowledge with regard to taxonomic diversity and distribution in Northern Hungary is based on sporocarp studies (Bohus and Babos, 1960; Takács and Siller, 1980; Rimóczi, 1992, 1994; Tóth, 1999; Siller et al., 2002a,b, 2006; Albert and Dima, 2005; Egri, 2007; Pál-Fám et al., 2007; Rudolf et al., 2008; Siller, 2010; Siller and Dima, 2014). In addition, several taxa in the ECM genera *Humaria*, *Genea*, *Tomentella*, and *Tuber* were characterized morphologically and molecularly in beech root tip studies carried out in a protected montane beech forest reserve in the Bükk Mountains (Kovács and Jakucs, 2006; Erős-Honti et al., 2008; Jakucs et al., 2015). However, the diversity of fungi in Pannonian forests and environmental factors influencing their distribution remained scarcely known.

This study provides the first systematic characterization of ECM fungal communities in various Pannonian forest types representative of the Bükk Mountains that were described coenologically in previous studies (Vojtkó, 2002; Borhidi, 2003; Vojtkó et al., 2010; Bölöni et al., 2011). More specifically, we compare the richness and community composition of ECM fungi among zonal, topographic and edaphic forest types that represent communities of different elevation zones, slope aspects, and soil pH, respectively. We hypothesized that ECM fungal community composition would differ significantly among these forest types due to niche processes, such as environmental filtering (Hypothesis 1). Temperature, available moisture and edaphic factors, particularly pH, are well known drivers of ECM fungal community composition at various spatial scales (Voříšková et al., 2013; Suz et al., 2014; Tedersoo et al., 2014; Kutszegi et al., 2015; Baldrian, 2017; Glassman et al., 2017; Koizumi et al., 2018; Rosinger et al., 2018; Aučina et al., 2019; Geml, 2019; Větrovský et al., 2019; Koizumi and Nara, 2020; Hupperts and Lilleskov, 2022; Mandolini et al., 2022). Based on these and other global and regional studies, and because many dominant ECM host tree genera are distributed along a wide range of forest types, we expected that the diversity and distribution of ECM fungi at landscape-scale would strongly be influenced by the above-mentioned abiotic factors (Hypothesis 2). Moreover, we expected differences in habitat preference among ECM fungal genera, as well as among congeneric species, due to differences in life strategies and in their physiological optima with respect to soil moisture and soil pH that likely influence their competitive abilities (Hypothesis 3).

## Materials and methods

### Study area

**FIGURE 1:**
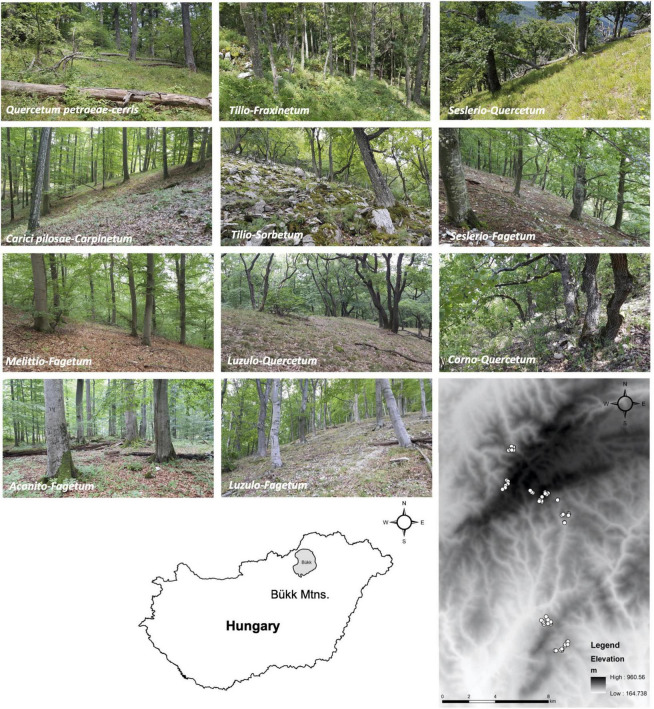
Illustrations of the sampled forest types and a map of the sampling localities, with the location of the region of study in Hungary. Full names and a brief ecological summary of the forest types are given in Table 1, while vegetation types, geographic coordinates, and abiotic variables corresponding to the sampling localities are listed in Supplementary Table 1.

The focal area of this study is located in the western half of the Bükk Mountains in northern Hungary, with elevation ranging from ca. 300 to 980 m a.s.l. (Figure 1). At lower elevations, the climate is subcontinental with mean January and July temperatures of ca. −3 and 20°C, respectively, and mean annual precipitation of ca. 550–580 mm, while at high elevations (i.e., between 800 and 980 m a.s.l.), mean annual temperature and precipitation values are 4–5°C lower 200–250 mm higher, respectively (Tóth, 1983; Horváth and Gaálová, 2007).

We sampled soil in 11 forest types of the Bükk Mountains, encompassing the diversity of mesoclimatic and edaphic conditions, represented by 62 sampling sites in mature stands in their most natural state possible. A brief ecological summary of the forest types is shown in Table 1, with geographic coordinates and environmental data of the 62 sampling sites shown in the Supplementary Table 1. Zonal forest types included Pannonian–Balkanic turkey oak–sessile oak forests (*Quercetum petraeae-cerris*) characteristic of gentle slopes at low elevations (200–450 m a.s.l.), Pannonian sessile oak–hornbeam forests (*Carici pilosae-Carpinetum*) in mesic, submontane (400–600 m a.s.l.) settings on gentle slopes and at valley bottoms, submontane beech forests (*Melittio-Fagetum*) on north-facing slopes between 400 and 750 m a.s.l., and montane beech forests (*Aconito-Fagetum*) in placor (near horizontal) settings and on gentle north-facing slopes above 750 m a.s.l. With respect to topographic forest types, we sampled thermophilous downy oak forests (*Corno-Quercetum pubescentis*) on shallow and rocky soil on steep (>20°) south-facing slopes with particularly warm and dry mesoclimate, dry to mesic limestone oak forests (*Seslerio-Quercetum*) on shallow, rocky soil of steep southern slopes at high elevations, mesic limestone beech forests (*Seslerio-Fagetum*) on shallow, rocky soil on northern and eastern slopes at mid- to high elevations, mesic linden-whitebeam rock forests (*Tilio*-*Sorbetum*) on shallow, rocky soils, on steep northern or eastern slopes, and mesic linden-ash rock forests (*Tilio*-*Fraxinetum*) on wind-swept mountain tops. Edaphic forest types included acidophilus oak (*Luzulo-Quercetum* or *Genisto tinctoriae-Quercetum*) and beech forests (*Luzulo-Fagetum*), both dry to mesic, found on acidic, shallow soils at elevations below and above 500 m a.s.l., respectively (Vojtkó, 2002; Borhidi, 2003; Vojtkó et al., 2010; Bölöni et al., 2011).

**TABLE 1:** Forest types included in this study with the main habitat characteristics and soil sampling localities.

Coenological forest type	Code	Habitat characteristics	Elevation range (m a.s.l.)
**Zonal forest types**			
*Quercetum petraeae-cerris*	Qpc	mesic to dry turkey oak–sessile oak forest in colline zone	250–400
*Carici pilosae-Carpinetum*	CpC	mesophilous sessile oak–hornbeam forest in submontane zone and in valleys	400–600
*Melittio-Fagetum*	MF	mesic submontane beech forest	400–750
*Aconito-Fagetum*	AF	mesic montane beech forest	above 750
**Topographic forest types**			
*Corno-Quercetum pubescentis*	CQ	dry, thermophilous downy oak forest on steep, south-facing slopes, and shallow, rocky soils	250–500
*Seslerio-Quercetum*	SQ	mesic to dry oak forest on shallow, rocky soils	400–700
*Seslerio-Fagetum*	SF	mesic beech forest on shallow, rocky soils, on northern or eastern slopes	above 500
*Tilio-Sorbetum*	TS	mesic whitebeam–linden forest on shallow, rocky soils, on steep northern or eastern slopes	above 500
*Tilio-Fraxinetum*	TF	mesic ash–linden forest on shallow, rocky soils, on mountain tops	above 500
**Edaphic forest types**			
*Luzulo-Quercetum* or *Genisto tinctoriae-Quercetum*	LQ	dry to mesic sessile oak forest on acidic soils	250–500
*Luzulo-Fagetum*	LF	dry to mesic beech forest on acidic, rocky soils	above 500

### Sampling and molecular work

At each site (ca. 10 × 25 m), 20 samples of top soil were taken from underneath the litter layer with a cylindrical soil corer (5 cm in diameter and 10 cm deep), and at least 2 m from each other. Soil samples collected at a given site were pooled, mixed and sieved (2 mm), resulting in a composite soil sample for each site. Approximately 20 g of each composite sample was kept frozen until DNA extraction, while the rest was used for soil chemical analyses to measure pH (water-based), and total carbon (*C*), nitrogen (*N*), phosphorus (*P*), and various micronutrient contents following Sparks et al. (1996).

Metagenomic DNA was extracted from 0.5 ml of soil from each composite sample using NucleoSpin^®^ soil kit (Macherey-Nagel Gmbh & Co., Düren, Germany), according to manufacturer’s protocol. The PCR and DNA sequencing were carried out as described in detail in Geml et al. (2014b). Briefly, primers fITS7 (Ihrmark et al., 2012) and ITS4 (White et al., 1990) with Illumina adapters were used to amplify the ITS2 region (ca. 250 bp) of the nuclear ribosomal rDNA repeat, using the following PCR conditions: one cycle of 95°C for 5 min, then 37 cycles of 95°C for 20 s, 56°C for 30 s, and 72°C for 1.5 min, ending with one cycle of 72°C for 7 min. A negative control consisting of MQ water instead of DNA were made and underwent the PCR reaction under the same experimental conditions, and were shown on a gel to be amplicon-free. In order to link the sequences to the sample source, forward and reverse primers were tagged with a combination of two different eight-nucleotide labels, resulting in a unique combination for each sample. The amplicon libraries were normalized for DNA concentration and were sequenced using Illumina NovaSeq at BaseClear (Leiden, Netherlands) to generate 250-bp paired-end reads with the default positive and negative controls routinely used by the sequencing company.

### Bioinformatic work

Raw DNA sequences were processed with the *dada2* package (Callahan et al., 2016), implemented in R v. 3.6.3 (R Development Core Team, 2015), designed to resolve fine-scale DNA sequence variation with improved elimination of artifactual sequences. Because *dada2* does not involve clustering sequences into OTUs and is robust for removing spurious data, the output of unique amplicon sequence variants (ASVs) captures both intra- and interspecific genetic variation of fungi found in the samples. This allows for the exploration of strain-level differences in inter- and intraspecific interactions. Raw sequences were truncated to 240 bp for forward and 200 bp for reverse to maintain an average Phred score of >30, denoised, chimera filtered, merged, and clustered into sequence variants. The maximum number of expected errors (maxEE) allowed in a read was 2. In order to minimize false presences, only ASVs with at least 10 sequences in a given sample were considered “present” in that sample. In addition, ASVs that occurred in only one sample were excluded from further analyses to avoid artifactual ASVs (Lindahl et al., 2013). After the above steps of quality filtering, there were 10,258 fungal ASVs from 539,139 ± 97,403 (mean ± SD) assembled fungal sequences per sample. The fungal community matrix was normalized (rarefied) by random subsampling to the smallest library size (277,881 reads) on a per-sample basis. Taxonomic assignments of fungal ASVs were made based on the UNITE reference database of representative sequences of all fungal species hypotheses (SHs) based on a dynamic delimitation (Kõljalg et al., 2013), using USEARCH v. 11 (Edgar, 2010). Selection of ECM fungal ASVs were made based on genus-level identification, with >90% sequence similarity, using the FungalTraits reference database (Põlme et al., 2020). ECM fungal ASVs were assigned to phylogenetic lineages of ECM fungi *sensu* Tedersoo and Smith (2013) based on the assignation of the matching SHs in UNITE. All sequences of ECM fungal ASVs analyzed in this paper have been submitted to GenBank (OP042390-OP043852).

### Geospatial informatics

For each sampling site, we obtained geographic coordinates and elevation data using a hand-held GPS device. We estimated insolation for each sampling site using the ArcGIS v. 10.4.1 Area Solar Radiation tool. This tool uses latitude of the site for calculations of solar declination and solar position, with correction of the radiation arriving at the surface based on a digital elevation model (DEM) to prepare a radiation raster with units of watt hours per square meter (WH/m^2^).

Because slope aspect and slope angle are known to influence mesoclimate, soil moisture, relative humidity, and soil chemical processes (McCune and Keon, 2002; Dobos, 2010; Gilliam et al., 2014; Méndez-Toribio et al., 2016), we accounted for the effect of slope aspect and slope angle, obtained from the DEM, and cross-checked with field measurements, as follows. When aspect is treated as a continuous variable from 0° to 360°, the two extreme values of this interval refer to the same slope aspect (north-facing). Therefore, we expressed aspect as northerly aspect following Calef et al. (2005) and Geml (2019), to better reflect the well-known environmental differences between north- and south-facing slopes, with values ranging from south = −90° to north = 90°. In addition, because high slope angle exacerbates the effect of slope aspect, we used the product of slope aspect and slope angle as a combined topographic variable, in addition to elevation.

We also calculated the Huglin heat sum index (or Huglin index) for all sampling sites, based on the input data obtained from the FORESEE v. 4.0 database, which is an open-access meteorological database that contains daily maximum/minimum temperature and precipitation data for Central Europe at 0.1 × 0.1° spatial resolution for the 1951–2021 time period based on the HUCLIM gridded dataset of the Hungarian Meteorological Service (HMS).^1^ The Huglin index, which was originally developed to characterize the mesoclimatic conditions of vineyards, is calculated as a product of the coefficient *K*, which depends on the latitude, and the sum of the arithmetic mean of daily mean- and daily maximum temperatures relative to the baseline temperature of 10°C from April 1 through September 30 (Huglin, 1986).

### Statistical analyses

Unless otherwise noted, all statistical analyses were carried out in R. We statistically compared ASV richness and relative abundance of ECM fungal genera among the samples with ANOVA and Tukey’s HSD test. ASV richness values were graphically presented as boxplots using the *ggplot2* R package (Wickham, 2016). Correlations among the ASV richness values of the five most diverse ECM fungal lineages and abiotic environmental variables were tested using quadratic regressions, which were visualized with *ggplot2*. Compositional differences among samples were visualized using non-metric multidimensional scaling (NMDS) in the *vegan* R package (Oksanen et al., 2012) with Bray–Curtis distance measure on the Hellinger-transformed matrix. We performed permutational multivariate analysis of variance (PERMANOVA) (adonis) in *vegan* to estimate the amount of variation explained by forest type, as categorical variable, and by edaphic and climatic factors as continuous variables. In addition, we performed indicator species analysis (Dufrêne and Legendre, 1997) with the *multipatt* function in the *indicspecies* package (De Cáceres et al., 2012) in order to identify characteristic and differential taxa for forest types. Finally, to better understand the influence of abiotic factors on the habitat preference of indicators and to illustrate species-level ecological differences within genera, we explored relationships between read abundance of indicator species and selected environmental variables. Specifically, we used linear regressions to correlate the rarefied read counts of indicator species in three ECM lineages with the highest number of indicators (/cortinarius, /inocybe, and /russula-lactarius) with five selected environmental variables that capture most of the edaphic and mesoclimatic differences among the sampling sites (pH, soil moisture, elevation, northerly aspect, and the Huglin index).

## Results

### Ectomycorrhizal fungal richness patterns

**FIGURE 2:**
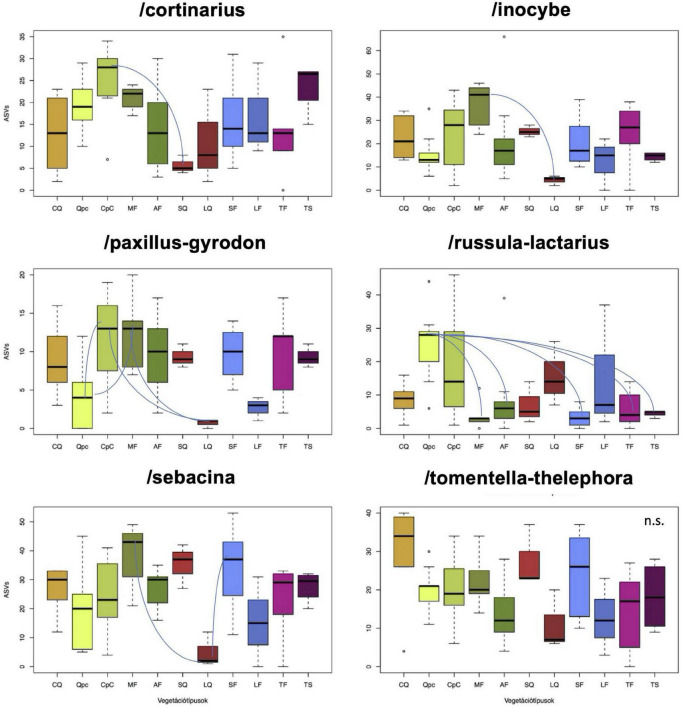
Comparison of amplicon sequence variant (ASV) richness of the dominant ectomycorrhizal (ECM) fungal lineages across among the sampled forest types. Means were compared using ANOVA and Tukey’s HSD tests, with arches denoting significant differences. Forest types are described in detail in the text, with abbreviations provided in Table 1.

Ectomycorrhizal fungi were represented by 3,759,052 DNA sequences that were grouped into 1,463 ASVs belonging to 58 genera and 39 phylogenetic lineages, mostly belonging to Basidiomycota. Of these, the twenty most ASV-rich lineages were/inocybe (239 ASVs), /sebacina (232), /tomentella-thelephora (201), /cortinarius (194), /russula-lactarius (129), /paxillus-gyrodon (47), /clavulina (39), /tuber-helvella (37), /hebeloma-alnicola (33), /cenococcum (29), /hysterangium (28), /genea-humaria (23), /boletus (20), /*elaphomyces* (19), /pseudotomentella (18), /piloderma (17), /marcellina-peziza (16), /sphaerosporella-wilcoxina (14), /hygrophorus (14), /hygrophorus (11), /amanita (9). There were substantial differences among the phylogenetic lineages with respect to patterns of ASV richness among the forest types. For example, /cortinarius was most diverse in the mesic sessile oak–hornbeam forests (*Carici pilosae-Carpinetum*) and least diverse in the limestone oak forests (*Seslerio-Quercetum*), while the/inocybe lineage was represented by the highest number of ASVs in the submontane beech forests (*Melittio-Fagetum*) and had the lowest number of ASVs in the acidophilous oak forests (*Luzulo-Quercetum*) (Figure 2). Somewhat similar trend was observed on the/paxillus-gyrodon lineage, where oak–hornbeam and submontane beech forests had significantly more ASVs than the acidophilous oak and the turkey oak–sessile oak (*Quercetum petraeae-cerris*) forests, with rest of the forest types showing intermediate ASV richness. Conversely, the /russula-lactarius lineage the most ASV-rich in the relatively warm turkey oak–sessile oak forests and had the fewest ASVs in the cool submontane beech, montane beech (*Aconito-Fagetum*), limestone beech (*Seslerio-Fagetum*), and whitebeam-linden (*Tilio-Sorbetum*) forests. The /sebacina lineage was most diverse in beech-dominated forests, while the /tomentella-thelephora lineage was comparably diverse in all forest types, although showed the highest richness in the thermophilous downy oak (*Corno-Quercetum pubescentis*) forests (Figure 2).

**FIGURE 3:**
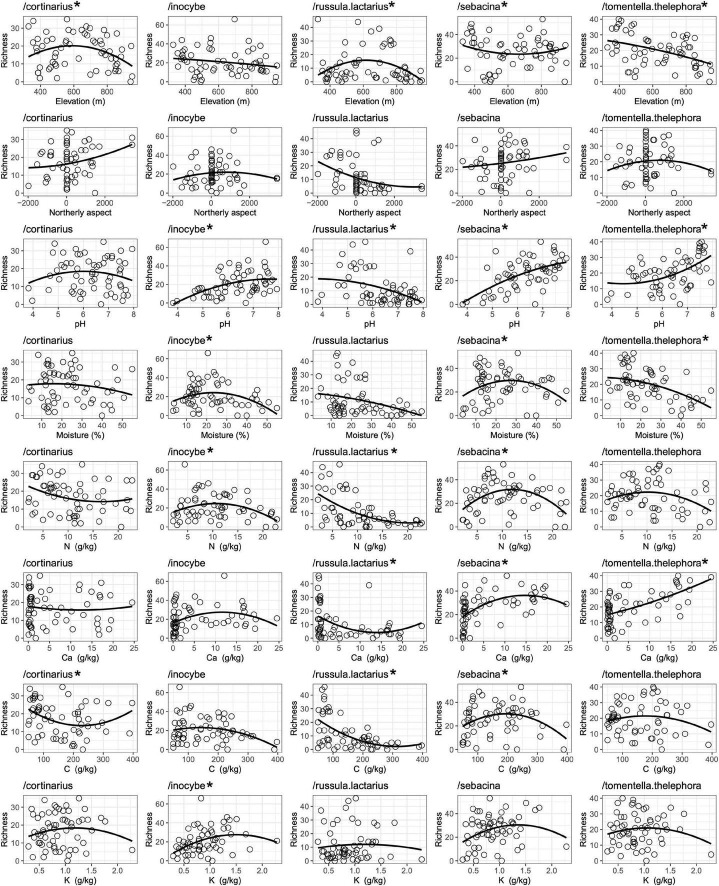
Correlations between amplicon sequence variant (ASV) richness of the dominant ectomycorrhizal (ECM) fungal lineages and selected environmental variables listed in Supplementary Table 1. Asterisk (*) indicates significant (*p* ≥ 0.05) correlation.

Regression analyses revealed different trends among ECM fungal genera with respect to relationships between ASV richness and various environmental variables. For example, richness of /cortinarius and /russula-lactarius was highest in mid-elevation, around 600 m a.s.l., while richness values of /tomentella-thelephora showed a strong monotonic decrease with increasing elevation (Figure 3). Northerly aspect did not show a significantly strong correlation with richness in the dominant fungal lineages, although we observed a weak positive relationship in /cortinarius and /sebacina and a weak negative relationship in the case of /russula-lactarius with northerly aspect. Soil pH showed strong positive correlation with richness in /inocybe, /sebacina, and /tomentella-thelephora, while negative correlation was observed with /russula-lactarius. The /inocybe and /sebacina lineages had the highest richness values at medium soil moisture levels, while /tomentella-thelephora correlated negatively with soil moisture. We observed negative correlation between richness in /russula-lactarius and soil *N* and *C* content, with /inocybe and /sebacina showing richness peaks at intermediate values. With respect to *K* content, only /inocybe showed a significant relationship, which was positive (Figure 3). Although not among the dominant lineages overall, the two most ASV-richness ascomycete lineages both correlated negatively with soil moisture: /cenococcum (*r*^2^ = 0.275, *p* < 0.0001) and /tuber-helvella (*r*^2^ = 0.1374, *p* < 0.0089) and with elevation: /cenococcum (*r*^2^ = 0.241, *p* = 0.0003), and /tuber-helvella (*r*^2^ = 0.0741, *p* = 0.0481).

### Ectomycorrhizal fungal community composition

**FIGURE 4:**
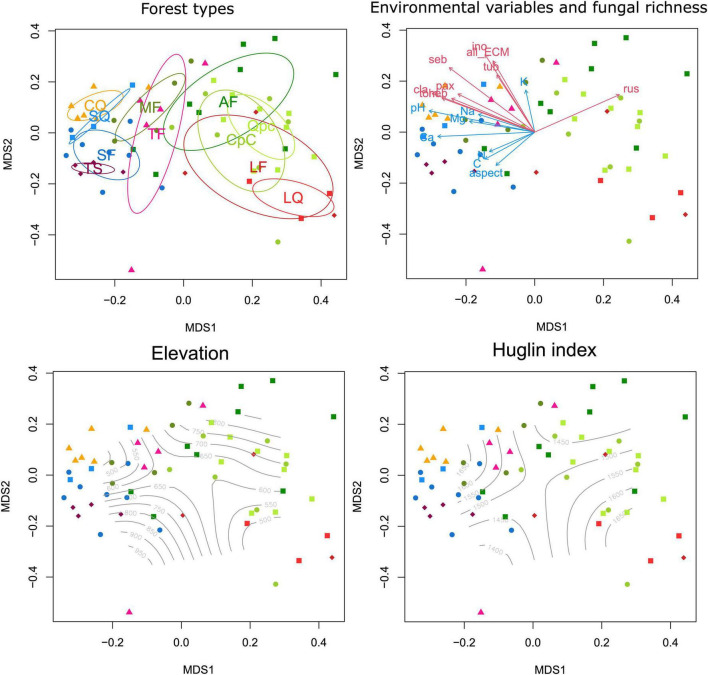
Non-metric multidimensional scaling (NMDS) ordination plot (final stress: 0.15734) of the total ectomycorrhizal (ECM) fungal community in the sampled forest types based on Hellinger-transformed data. Ellipses indicating standard deviation of compositional differences of forest types, vectors of environmental variables and richness values of ECM fungal lineages showing significant correlations with ordination axes, and isolines of elevation and Huglin index are displayed in four identical ordination plots.

Community composition of ECM fungal communities was strongly structured among Pannonian forests, mostly driven by soil pH and other edaphic factors (Figure 4). Soil pH (*r*_NMDS1_ = −0.9602, *p* < 0.0001), *C* content (*r*_NMDS1_ = −0.8181, *p* < 0.0001), and concentrations of cations, such as Ca (*r*_NMDS1_ = −0.9989, *p* < 0.0001), Mg (*r*_NMDS1_ = −0.9709, *p* < 0.0001), and Na (*r*_NMDS1_ = −0.9155, *p* < 0.0001) correlated strongly with the first axis.

PERMANOVA analyses confirmed that ECM fungal community composition was strongly structured by coenological forest type, explaining 28.06% of compositional differences among all samples (Table 2). With respect to edaphic variables, pH, soil moisture and Ca, *C*, *K*, and *P* content contributed significantly to the combined model after accounting for correlations among variables. Of these, pH explained more than 9% and Ca explained 3.42% of the compositional variance, with the rest explaining less than 3%. Of the mesoclimatic and topographic variables, the Huglin index and northerly slope aspect remained significant, explaining 2.14% of variance (Table 2).

**TABLE 2:** Proportions (%) of compositional variance explained by environmental variables in individual models based PERMANOVA, with variables providing significant, unique contribution to the combined model indicated in bold.

	All ECM fungi	/Cortinarius	/Inocybe	/Russula-lactarius	/Sebacina	/Tomentella-thelephora
**Categorical variable**						
Forest type	28.059	27.803	24.721	28.275	25.488	29.102
**Continuous variables**						
pH	**9.004**	**8.352**	**6.754**	**6.980**	**5.963**	**10.440**
*C*	**4.674**	**4.422**	**4.060**	**5.932**	**4.279**	**3.933**
*N*	4.083	4.218	3.450	5.341	3.315	3.353
CN	1.919	2.282	1.348	1.051	1.745	2.512
Na	4.071	4.605	3.358	**4.783**	2.491	3.788
*K*	**2.950**	3.065	**4.319**	**2.931**	**2.623**	**3.115**
Ca	**8.380**	**7.773**	**6.329**	**8.202**	**7.528**	**9.356**
Mg	4.865	4.441	4.016	6.188	3.484	5.620
*P*	**2.486**	2.441	2.167	2.266	2.943	2.722
Huglin index	**3.875**	2.600	2.445	2.844	**3.175**	**2.769**
Soil moisture	**3.655**	**3.385**	**2.114**	**4.578**	**2.503**	**3.199**
Elevation	3.444	2.797	2.637	3.317	3.899	3.555
Northerly aspect	**3.438**	3.639	3.019	**4.349**	2.768	**2.617**

When the dominant ECM fungal lineages were analyzed separately, forest types always had a significant correlation with community structure, explaining between 24.72 and 29.1% of the variance. After accounting for correlations among environmental variables, soil pH, Ca and *C* content, and soil moisture contributed to the combined model, explaining significant proportions of the community composition in all tested lineages and *K* content in all except/cortinarius. Northerly aspect remained significant to explain part of the compositional variance in /russula-lactarius, /tomentella-thelephora, and in the total ECM fungal community, while the contributions of the Huglin index and soil *P* content were only significant in the combined model in total ECM community (Table 2).

**FIGURE 5:**
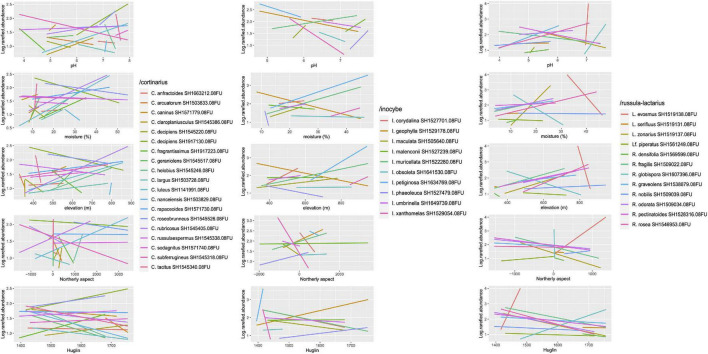
Correlations between selected environmental variables and log-transformed rarefied read abundance of indicator species of various forest types in three dominant ectomycorrhizal (ECM) fungal lineages. The slopes and the positioning of the regression lines along the *x* axis suggest species-level ecological differences within genera. The full list of indicator species can be found in Supplementary Table 2.

One hundred thirty-seven ECM fungal ASVs were significant (*p* < 0.05) indicators of a certain coenological forest type. Of these, thermophilous downy oak forests and oak–hornbeam forests had the highest number of indicators (18 ASVs each), followed by submontane beech and whitebeam–linden forests (16 each), turkey oak–sessile oak and limestone oak forests (15 each), and acidophilous oak forests (11), the rest having less than 10 indicators (Supplementary Table 2). In most ECM fungal genera, ASVs assigned to different SHs tended to be indicators for distinct forest types, particularly when the sequence similarities were high (above 99%). As expected from the indicator species analysis, we found strong species-level differences within all the selected phylogenetic lineages with respect to their correlations with the tested environmental variables. These differences were partly directional (positive or negative correlation) and partly were differences in range breadth and overlap with respect to the given variable (Figure 5). For example, some indicator species occurred along a wide range of pH, moisture or elevation, while others seemed more restricted. In the latter group, we found indicators that seemed restricted to acidic vs. non-acidic or alkaline pH, dry vs. mesic soil or low vs. high elevation. Interestingly, some indicator species had relatively wide occupied range for some variables and a narrow range for others (e.g., *Inocybe obsoleta*, *Russula globispora*) (Figure 5).

## Discussion

Our study, which is the first to characterize ECM fungal communities across a wide range of Pannonian forest types that span mesoclimatic and edaphic gradients, clearly show that (1) the composition of ECM fungi differ among coenological forest types; (2) the diversity and distribution of ECM fungi on the landscape are driven primarily by edaphic factors, such as soil pH, Ca and *C* content and soil moisture, as well as by temperature; (3) there are important ecological differences among ECM fungi at fine taxonomic scales with regard to habitat preference, as suggested by the indicator species and the pronounced differences among genera as well as congeneric species with respect to their relationships with abiotic variables. These key findings highlight various components within the conceptual framework of community assembly, the non-stochastic component of which likely is driven, at least in part, by species-level niche differences as well as by differences in competitive abilities under certain environmental conditions. Together, these are expected to contribute to environmental filtering *sensu* Chesson (2000) that, in this sense, includes not only the exclusion of species that are not able to survive under particular abiotic conditions, but also the role of the abiotic environment in coexistence dynamics.

It is reassuring to see that the forest types detailed above, formerly described based on detailed coenological studies of woody plants, forbs, graminoids as well as non-vascular plants (Vojtkó, 2002; Borhidi, 2003; Vojtkó et al., 2010; Bölöni et al., 2011), seem to capture well the consistent and largely predictable mesoclimatic and edaphic differences driven by topography and geology. It is noteworthy, that although ECM fungi are associated with the dominant trees that form the above forest types and are widely distributed throughout the landscape, the coenological associations are mostly differentiated based on understory non-ECM plants that seem to reflect better the edaphic and mesoclimatic conditions prevailing at the sites. Based on their above-mentioned connection with the ECM fungal community, the delimitation and mapping of Pannonian forest types provide useful tools to better understand the distribution of ECM fungi at landscape scales in this biogeographic region.

In our study, we show examples of profound differences among congeneric ECM fungal species with respect to their preference to abiotic factors as well as their niche breadth regarding the variable in question.

The role of soil pH in shaping fungal communities has been widely documented at various spatial scales (Coughlan et al., 2000; Lauber et al., 2008; Rousk et al., 2010; Geml et al., 2014a,b; Tedersoo et al., 2014; Kutszegi et al., 2015; Glassman et al., 2017; Rosinger et al., 2018; Geml, 2019; Větrovský et al., 2019). Beside the geological parent material, soil pH is often influenced by mesoclimatic factors, such as temperature and soil moisture, with soils exposed to lower temperatures and higher moisture generally having lower pH than warmer and dried soils on the same landscape (Geml et al., 2014b; Gilliam et al., 2014; Chu et al., 2016). Such mesoclimatic differences affecting soil characteristics could be due to differences elevation or to slope aspect, both of which influence surface temperature, soil moisture, relative humidity, and soil chemical processes (McCune and Keon, 2002; Fekedulegn et al., 2003; Dobos, 2010; Gilliam et al., 2014; Méndez-Toribio et al., 2016). In fact, recent studies have revealed the role of slope aspect to shape fungal communities at landscape scales at a different locality in northern Hungary (Geml, 2019) and in central Asia (Chu et al., 2016). Elevation is also known to influence fungal community composition (Coince et al., 2014; Geml et al., 2014b,^2017^, ^2022^; Javis et al., 2015; Wicaksono et al., 2017), although in this study, the effect of elevation was not significant in the combined model, likely because of the tight relationship with temperature (Huglin index), which had somewhat higher correlation with community composition when environmental variables were analyzed separately.

The influential roles of soil moisture and Ca in shaping fungal communities, other than their influence on pH, observed in our study are in agreement with what has been found in the global soil fungal community study of Tedersoo et al. (2014). Calcium plays crucial roles in numerous physiological processes related to growth and stress responses (McLaughlin and Wimmer, 1999) and shapes plant and animal communities (Beier et al., 2012). Because of its low mobility, the availability of Ca often poses limitations on forest structure and function, particularly in dry and acidic soils. The roles of Ca, that are particularly relevant to ECM fungi, include effects on the structure and function of plant cell membranes that influence nutrient uptake by roots and fluxes through leaf membranes, the transport of carbohydrates from leaves to other plant parts, such as roots that directly interact with mycorrhizal fungi, litter decomposition rates, and the formation of humus and soil aggregates (McLaughlin and Wimmer, 1999).

Similarly, *C* and *N* contents are among the most important edaphic variables globally that influence richness and community composition in fungi (Tedersoo et al., 2014). Although both *C* and *N* content correlated significantly with richness values in three out of five ECM fungal lineages, the strong contribution of *C* content to explain community composition in all dominant lineages and the lack of significance of *N* content in the combined model seems unexpected. Because *N* content correlated significantly with fungal community composition in all lineages when analyzed separately, it is likely that its lack of unique contribution to the combined model is because of a strong correlation between *N* and *C* contents (*r*^2^ = 0.8398, *p* < 0.0001).

Although the exact mechanisms are yet to be elucidated, it is likely that the chemical differences among the sampling sites influence the competitive dynamics of fungi and, thus, they represent environmental filters with regard to establishment and persistence in the community (Chesson, 2000; Lennon et al., 2012). The observed correlations of the richness and composition with edaphic factors suggest that some effects on the ECM fungal community could be direct, e.g., physiologically constraining, while most effects may be indirect, e.g., *via* nutrient availability and nutrient acquisition capabilities. Despite the relatively high proportions of compositional variance explained by the tested environmental variables, the proportion of residual variance not explained by the above variables still exceeded 60%, indicating that there likely are other abiotic or biotic factors not tested in this study, e.g., soil bacterial and faunal communities, that could affect ECM fungal community composition. In addition, although not directly tested in this study, neutral (stochastic) processes, e.g., dispersal, priority effect, and drift, likely contribute to the observed compositional differences of ECM fungal communities among sampling sites, as widely reported for diverse biological communities (Vellend, 2010; Rosindell et al., 2012; Vellend et al., 2014).

Beside the ecological information obtained, this study also provides the first overview of taxonomic diversity and identity of ECM fungi in the dominant Pannonian forest types based on soil DNA data. The full list of fungal SHs that matched an ECM fungal sequence generated from soil samples in the Pannonian forest types in this study and taxonomic classification (Supplementary Table 2) will facilitate future mycological and fungal ecological studies in the region. The 1,463 ASVs representing 58 ECM fungal genera indicates high diversity, although the true diversity of ECM fungi in the region certainly is considerably higher, because many species known to occur in the sampling region based on sporocarp studies were not found in the soil samples. Although this could partly be due to primer biases against lineages with longer PCR amplicons (Baldrian, 2019), it is not surprising based on the high spatial patchiness of soil fungi (Branco et al., 2013; Cale et al., 2021) and the random nature of soil sampling, which is particularly true for diverse lineages that are well represented in sporocarp-based studies, e.g., /amanita, /boletus, and /russula-lactarius, but are relatively underrepresented in soil DNA due to their low mycelial biomass in soil compared to other ECM genera (Gardes and Bruns, 1996; Geml et al., 2012). In these groups, in order to capture their total diversity, many more samples will be needed across the study region. On the other hand, our data provides unprecedented insight into ECM fungal groups with inconspicuous fruiting bodies, e.g., /sebacina and/tomentella-thelephora that represent two of the dominant fungal lineages and that generally are underrepresented in sporocarp surveys (Gardes and Bruns, 1996; Kõljalg et al., 2000; Geml et al., 2012). In addition, the data presented provides novel information on the taxonomic diversity and distribution of hypogeous ECM fungi, including several unidentified *Elaphomyces*, *Gautieria graveolens*, *Genea arenaria*, *G. dentata*, and *G. verrucosa*, *Hymenogaster citrinus*, *H. griseus*, *H. huthii*, *H. luteus*, and *H. rehsteineri*, *Hysterangium calcareum*, *Hy. nephriticum*, *Hy. pompholyx*, and *Hy. stoloniferum*, *Melanogaster ambiguus*, *M. broomeanus*, *M. spurius*, and *M. variegatus*, *Scleroderma areolatum*, *Tuber aestivum*, *T. borchii*, *T. brumale*, *T. excavatum*, *T. fulgens*, *T. puberulum*, *T. rapaeodorum*, and *T. rufum*, and *Wakefieldia macrospora*. Most of these species have been recorded in Hungary in sporocarp studies, except for *G. graveolens*, as this genus is only represented by an unidentified species in the list of hypogeous fungi for the Carpathian–Pannonian region compiled by Bratek et al. (2013). In addition, soil DNA studies, such as this, can provide valuable spatial data for mapping the distribution of rare, protected ECM fungi and provide valuable information on their habitat preference. For example, we found the protected *Strobilomyces strobilaceus* in soil from a sessile oak–hornbeam forest and from an acidophilous oak forest, which is in agreement in terms of habitat preference with sporocarp records of this species from mesic acidic forests in Hungary (Siller et al., 2006). The spatial data presented in this paper complement sporocarp-based assessments and highlights the potential of DNA-based characterization of fungal communities in biological monitoring and conservation of fungi. In addition, the ecological data on the abiotic factors influencing the diversity and distribution of various ECM fungi on the landscape provide a baseline data for climate change studies as well as inform us about possible responses of ECM fungal communities to climate change. This is particularly relevant for the sustainable management of our natural resources, as ECM fungi are vital symbionts of the dominant trees in Pannonian forests and contribute to tree health by providing water and nutrients and mitigating abiotic stresses. The habitat specificity and rapid reaction of soil fungi to changes in environmental factors provides us with the possibility of detecting trends in forest dynamics early and take action accordingly to maintain diverse and resilient forest ecosystems with diverse ecosystem functions.

## Data availability statement

The data presented in this study are deposited in the GenBank repository accession numbers: OP042390–OP043852.

## Author contributions

JG and JS conceived the research plan and did the field sampling in 2018. JS arranged the chemical analyses of the soil samples. CL and JG generated the DNA data from the soil samples. RN did the geospatial analyses to obtain insolation and Huglin index data for the sampling sites and prepared the maps for Figure 1. JG did all bioinformatic and statistical analyses and wrote the first draft of the manuscript. All authors provided feedback on the first draft and participated in finalizing the manuscript.

## Footnotes

1
http://nimbus.elte.hu/FORESEE/
